# Circulating Fibroblast Growth Factor 21 Is A Sensitive Biomarker for Severe Ischemia/reperfusion Injury in Patients with Liver Transplantation

**DOI:** 10.1038/srep19776

**Published:** 2016-01-25

**Authors:** Dewei Ye, Huating Li, Yudong Wang, Weiping Jia, Jian Zhou, Jia Fan, Kwan Man, Chungmau Lo, Chiming Wong, Yu Wang, Karen S.L. Lam, Aimin Xu

**Affiliations:** 1 State Key Laboratory of Pharmaceutical Biotechnology, The University of Hong Kong, Hong Kong, China; 2 Department of Medicine, The University of Hong Kong, Hong Kong, China; 3 Department of Pharmacology & Pharmacy, The University of Hong Kong, Hong Kong, China; 4 Department of Endocrinology and Metabolism, Shanghai Jiao Tong University Affiliated Sixth People’s Hospital, Shanghai, China; 5 Department of Liver Surgery, Liver Cancer Institute, Zhongshan Hospital, Fudan University, Shanghai, China; 6 Department of Surgery, The University of Hong Kong, Hong Kong, China

## Abstract

Hepatic ischemia/reperfusion (I/R) injury is a major cause of morbidity and mortality after liver surgery. Therefore, it is important to identity reliable biomarkers to assist early diagnosis of hepatic I/R injury. This study aimed to investigate the potential of serum levels of fibroblast growth factor 21 (FGF21) as a biomarker for hepatic I/R injury in patients with liver transplantation. Two independent cohorts of liver transplantation patients were recruited for determination of serum levels of FGF21, ALT, and AST. The results demonstrated that serum FGF21 at 2 hours post-reperfusion in cohort-1 exhibited an approximately 20-fold elevation relative to those in healthy subjects. In blood samples dynamically collected in cohort-2, a dramatic increase in serum FGF21 levels (~25-fold) was observed at two hours after surgery, whereas the peak levels of serum ALT and AST were detected only after 24 hours. Temporal correlation analysis demonstrated a significant association of peak serum levels of FGF21 at 2 hours with the magnitude of the increase in both serum ALT and AST levels at 24 hours post transplantation. In conclusion, serum FGF21 may represent a sensitive and specific prognostic biomarker for early detection of I/R injury in patients with liver transplantation.

Hepatic ischemia/reperfusion (I/R) injury is a frequently encountered problem in close association with a number of clinical conditions, including liver transplantation, Pringle maneuver during tumor resection, liver trauma, veno-occlusive disease, hemorrhagic shock–resuscitation, and heart failure^1^,^2^. Hepatocellular damage in hepatic I/R injury is initiated by the interruption of blood flow into the liver and further amplified by the subsequent reperfusion-medicated dramatic release of reactive oxidative species and sterile inflammation^3^. In particular, hepatic I/R injury in liver transplant recipients has a significant impact on acute liver failure/graft rejection, remote organ injury, and even the increased rate of mortality^2^,^4^. Current therapeutic interventions exhibit limited efficacy on preventing or treatment of this clinical syndrome.

Despite the clinical significance, detection and early diagnosis of hepatic I/R injury still remains a challenging task, in part due to the lack of reliable biomarkers with high sensitivity and specificity. In the clinic, serum level of alanine aminotransferase (ALT) and aspartate aminotransferase (AST), the enzymes released into the circulation following the loss of membrane integrity, is the most commonly used biomarker for both acute and chronic liver injury^5^,^6^. However, the use of ALT to assess liver injury has several limitations. First, serum ALT levels do not always correlate well with the severity of liver damage. This is exemplified by the increase in serum ALT levels caused by several drugs, including tacrine for Alzheimer’s disease^7^, statins for hypercholesterolemia^8^, as well as heparin for venous thromboembolic disease^9^. Second, the elevation of circulating ALT is not specific to liver injury, and it can also be released from other organs such as heart and skeletal muscle^10^. In this context, the identification and development of additional circulating biomarkers specific to liver function should be crucial for the early detection of hepatocellular damage and subsequent therapeutic interventions. Several candidate markers, including cytokines^11^, high mobility group box 1 protein^12^, tissue-specific messenger RNAs^13^, and microRNAs^14^, have been reported. However, clinical utility of all these emerging markers still needs further validation.

Fibroblast growth factor 21 (FGF21) is a metabolic hormone secreted predominantly by hepatocytes^15^. Unlike the classical members of FGF family, FGF21 does not possess heparin-binding properties, enabling it to be released into the circulation^16^. The amino acid sequences of FGF21 are highly conserved in mammals (approximately 75% identity between mouse and human FGF21)^17^. Hepatic FGF21 expression is under the control of peroxisome proliferator activated receptor α (PPAR-α), a nuclear receptor that is activated under nutrient-deficient conditions such as prolonged fasting^18^. FGF21 exerts its actions by interacting with the receptor complex between FGF receptor (FGFR) and β-Klotho (KLB), thereby leading to activation of extracellular signal-regulated kinase 1 and 2 and other kinases^15^,^19^,^20^. In the liver, FGF21 induces the expression of genes involved in fatty acid oxidation and gluconeogenesis^21^,^22^,^23^, but suppresses the expression of genes involved in cholesterol biosynthesis^21^. A growing body of evidence from animal studies has demonstrated FGF21 as an important metabolic regulator with pleiotropic effects on glucose and lipid homeostasis^20^,^24^. Therapeutic administration of recombinant FGF21 exerts a variety of favorable effects in both obese rodents and nonhuman primates, including reduction of body weight, alleviation of hyperglycemia and insulin resistance, improvement in lipid profiles and hepatic steatosis^22^,^25^.

Despite its multiple beneficial effects on glucose and lipid metabolism, circulating levels of FGF21 are elevated in obese subjects and patients with type 2 diabetes^26^,^27^. Several cross-sectional studies on different ethnic groups have demonstrated a modest, but significant elevation of serum FGF21 in patients with non-alcoholic fatty liver disease (NAFLD)^28^,^29^. Furthermore, there is a strong positive correlation between serum FGF21 and circulating levels of both γ-glutamyltransferase and ALT, suggesting that FGF21 may be a potential biomarker for the diagnosis of NAFLD^28^,^30^. In rodent model of acetaminophen-induced hepatotoxicity, circulating levels of FGF21 exhibited a dramatic increase, which in turn acts as an adaptive response to protect against hepatocyte death by enhancing antioxidant capacity^31^. However, the association of FGF21 with hepatic I/R injury in liver transplantation has never been explored so far. The present study aimed to investigate the potential of serum FGF21 as a biomarker for I/R-induced liver injury in patients with liver transplantation.

## Results

### Patient Characteristics

The clinical characteristics of the liver transplantation patients in the two study cohorts are shown in Table 1. In cohort-1, a total of 38 subjects (30 males and 8 females) were enrolled. The primary disease for liver transplantation was hepatocellular carcinoma (68.4%) and hepatitis B virus-related cirrhosis (23.7%). The median levels of serum ALT and AST before liver transplantation are 44.5 U/L and 66.5 U/L, respectively. A total of 13 subjects (9 males and 4 females) were recruited in cohort-2. Among them, 12 subjects received liver transplantation for hepatocellular carcinoma. The median levels of serum ALT and AST immediately before liver transplantation are 42 U/L and 50 U/L, respectively.

### Serum FGF21 Levels Are Markedly Elevated at the Early Stage of Patients Receiving Liver Transplantation

**Figure 1:**
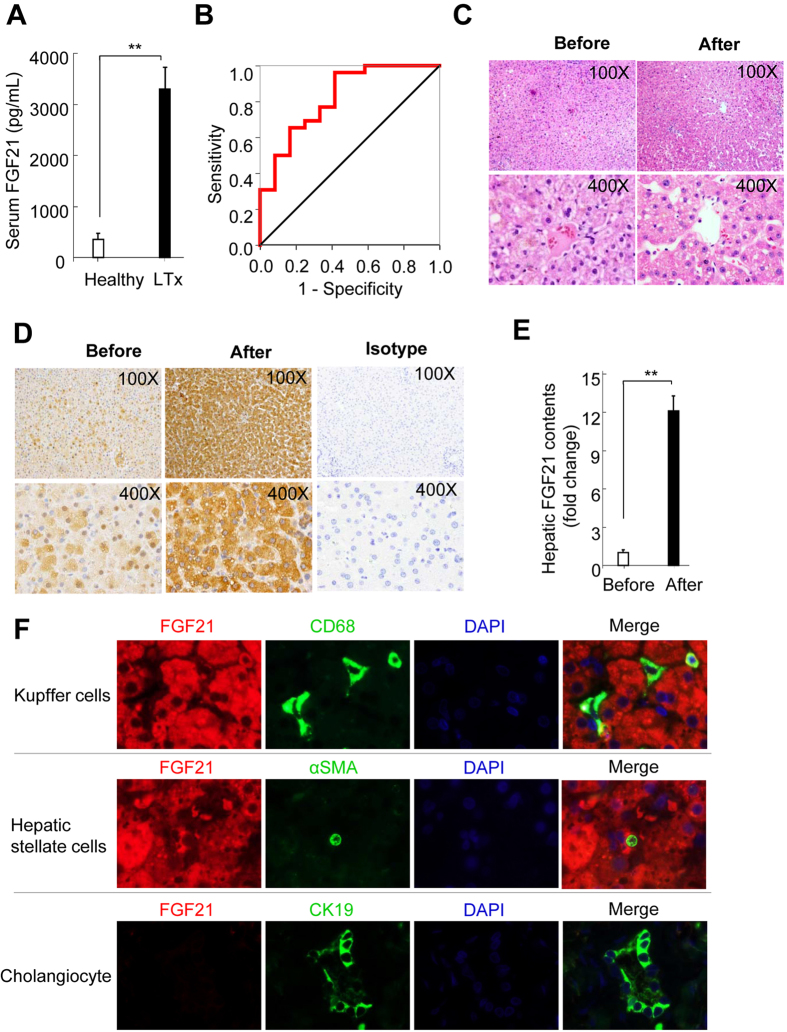
Circulating levels and hepatic expression of FGF21 were markedly increased in patients with liver transplantation. (**A**) Serum FGF21 levels in 38 patients receiving liver transplantation (LTx) and age-and sex-matched healthy subjects. Blood samples from LTx patients were collected at 2 hours after hepatic artery declamping. (**B**) Representative receiver operator characteristic (ROC) curve for serum FGF21 determined at 2 hours post reperfusion for the prediction of severe hepatic I/R injury (defined by serum levels of AST at day 1 post liver transplantation >240 U/L). (**C**) Representative micrographs of hamotoxylin & Eosin-stained liver sections from the same donor liver before and after LTx (Original magnification: 100x upper; 400x, lower). (**D**) Hepatic FGF21 levels determined by immunohistochemistry in the same liver sections as stated in panel B (Original magnification: 100x upper; 400x, lower). (**E**) Semi-quantification of FGF21-positive area in micrographs shown in panel C. (**F**) Co-immunofluorescence staining of FGF21 with Kupffer cell marker CD68 (upper), hepatic stellate cell marker α-smooth muscle action (α-SMA, middle), and cholangiocyte marker cytokeratin 19 (lower) in liver sections collected at 2 hours post-liver transplantation (Original magnification: 1000x). Quantitative data are expressed as mean ± S.D. ^**^P < 0.01.

We first measured FGF21 levels in serum samples collected at two hours after hepatic artery declamping in liver transplantation patients in cohort-1. Notably, serum FGF21 levels in liver transplantation patients exhibited an approximately 20-fold elevation, when compared with age- and sex-matched healthy individuals (3402.9 ± 436.6 pg/mL vs. 183.2 ± 39.8 pg/mL) (Fig. 1A). To explore the diagnostic performance of circulating FGF21 for the prediction of severe hepatic IR injury (defined by serum levels of AST at day 1 post liver transplantation >240 U/L (6 times upper limit of normal), receiver operating characteristics (ROCs) was calculated in study cohort 1. The results demonstrated that area under the ROC curve was 0.821, indicating a reliable performance of circulating FGF21 for the prediction of severe hepatic I/R injury. The optimal cut-off value of circulating FGF21 at 2 hours post-reperfusion for predicting severe hepatic I/R injury was 987.66 pg/mL as determined by Youden index. Therefore, serum FGF21 levels >987.66 pg/mL at 2 hours after liver transplantation predicted severe hepatic IR injury on the first postoperative day with a sensitivity of 96.2% (detection of true positive = detection of patients with serum AST levels >240 U/L) and with a specificity of 58.3% (1 – 0.417 = 0.583; false-positive rate 41.7%) (Fig. 1B).

To delineate the potential association between serum FGF21 levels and potential alterations in hepatic FGF21 production during the acute phase in liver transplantation, paired liver specimens collected from the same donor organ before and after reperfusion were subjected to hematoxylin and eosin staining. There is no evident alteration in liver morphology observed in both liver sections obtained before and after implantation (Fig. 1C), although it has been documented that oxidative stress and the activation of Jun amino-terminal kinase (JNK) occurred in the liver as early as 25 to 30 minutes post-reperfusion^32^. We next determined FGF21 protein levels by immunohistochemistry in paired liver sections collected from the same donor organ before and after implantation. In liver sections collected before its removal from donor subjects, less than 20% hepatocytes showed weak FGF21 positivity, whereas more than 90% of hepatocytes exhibited FGF21 positivity with significantly enhanced signal strength in liver tissue harvested after reperfusion (Fig. 1D,E).

To further investigate whether FGF21 was exclusively expressed in hepatocytes after liver transplantation, co-immunofluorescence of FGF21 with markers of non-parenchymal cells within the liver, including CD68 for Kupffer cells, α-smooth muscle actin for hepatic stellate cells, and cytokeratin 19 for cholangiocytes were performed. The results demonstrate that FGF21 signals did not co-localize with any of these markers of non-parenchymal cells (Fig. 1F). In addition, FGF21-positive signals mainly located in the cytoplasmic region, but was virtually undetectable in the nuclear area of hepatocytes. In light of a previous report demonstrating liver as the predominant source organ of systemic FGF21^15^,^31^,^33^, our data demonstrated that serum FGF21 levels were dramatically elevated at the very early stage during liver transplantation, which is mainly due to the marked increase in FGF21 production in hepatocytes in response to I/R during liver transplantation.

### Serum FGF21 Levels Exhibited a Distinct Dynamic Profile during Liver Transplantation

**Figure 2:**
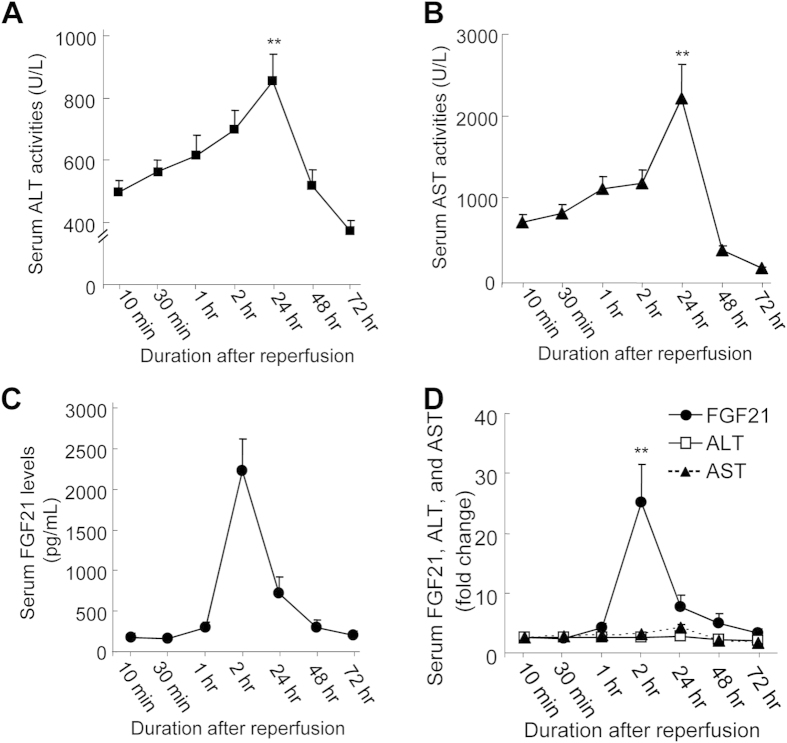
Temporal changes in serum levels of ALT, AST, and FGF21 during the first 72 hours after liver transplantation (LTx). Serum levels of ALT (**A**), AST (**B**), and FGF21 (**C**) were determined in blood samples collected at seven different time points (10 min, 30 min, 1 hr, 2 hr, 24 hr, 48 hr and 72 hr) after reperfusion in 13 patients with LTx. (**D**) Fold changes in serum ALT, AST, and FGF21 levels relative to their respective concentrations at 10 min n = 13. Data are expressed as mean ± S.D. ^**^P < 0.01 vs. 10 min.

The markedly elevated serum FGF21 levels at 2 hours post-reperfusion in cohort-1 prompted us to investigate the dynamic profiles of serum FGF21 levels during liver transplantation. To this end, we collected serum samples at various time points in 13 patients receiving liver transplantation in cohort-2. There was a marked elevation of both serum levels of ALT and AST as compared to baseline levels immediately after transplantation, followed by the second major rise of serum ALT and AST at 24 hours after surgery (Fig. 2A,B; Table 1). Intriguingly, serum FGF21 levels remained at a relatively low level (<300 pg/mL) during the first hour, but increased sharply to 2229.5 ± 389.7 pg/mL at 2 hours, then declined progressively to its baseline level at 48 hours after liver transplantation (Fig. 2C). Furthermore, the fold increases of serum FGF21 at its peak value at 2 hours were substantially higher than those of serum ALT and AST at their peak values at 24 hours after liver transplantation (Fig. 2D). Collectively, our data suggest that a marked elevation of serum FGF21 occurs well before massive hepatocellular damage induced by I/R injury during liver transplantation.

### Serum FGF21 Levels at Early Stage Significantly Correlate with the Amplitude of the Increase in Serum ALT and AST in Later Stage

**Figure 3:**
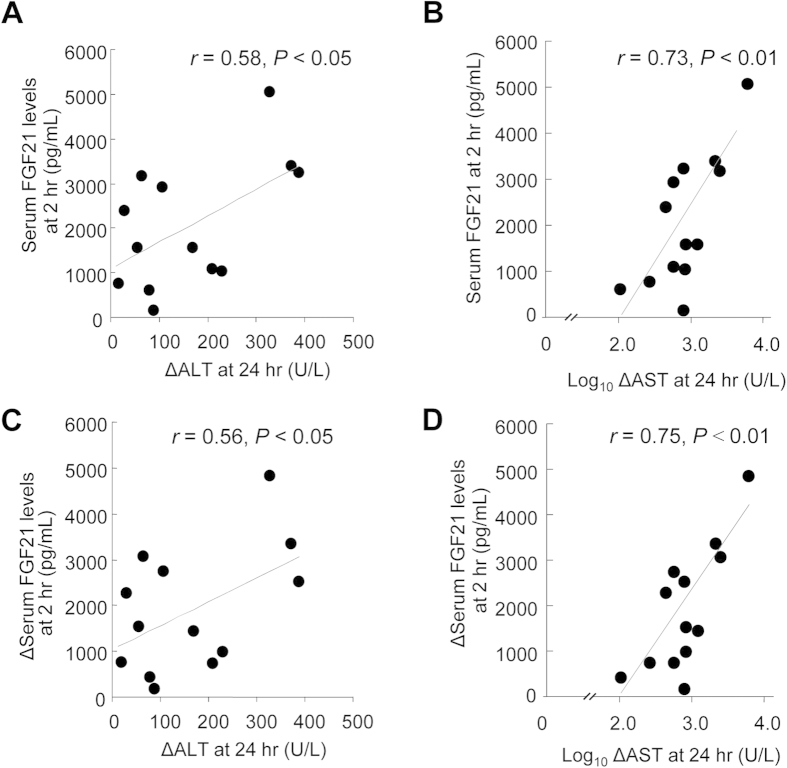
Association between changes in serum FGF21 levels and serum ALT and AST Levels in patients receiving liver transplantation. Serum levels of FGF21, ALT and AST were measured at different time points in 13 patients who received liver transplants as in Fig. 2. (**A,B**) Correlation between the peak serum FGF21 levels at 2 hours and the peak values in serum ALT (ΔALT) and logarithmically transformed data of AST (ΔAST) expressed as net increases from 10 min to 24 hours after liver transplantation (**C,D**) Correlation between the increase in serum FGF21 (ΔFGF21) from 10 min to 2 hours and the increase in serum ALT (ΔALT) and logarithmically transformed data of the increase in AST (ΔAST) from 10 min to 24 hours after LTx. n = 13.

Hepatic I/R injury exerts a profound and harmful impact on graft rejection or chronic liver dysfunction^2^,^4^. Hence, the prediction of the high risk for the development of hepatic I/R injury should be of significant importance for clinical management of I/R injury in liver transplantation. To further explore the temporal relationship between changes in serum FGF21 and the two classical markers of liver injury, we analyzed their association between the peak values of serum FGF21 at 2 hours and the peak levels of ALT and AST at 24 hours. This analysis demonstrated that serum FGF21 levels at 2 hours correlated positively with the magnitude of increase of both ALT (*r* = 0.58, *P* < 0.05) and AST (*r* = 0.73, *P* < 0.01) from 10 minutes to 24 hours after liver transplantation (Fig. 3A,B). In addition, the amplitudes in FGF21 increase at the first 2 hours post-reperfusion also showed significant and positive correlation with the extents in the increase of serum activities of both ALT and AST in day 1 (Fig. 3C,D), suggesting that the elevation in serum FGF21 levels at 2 hours may serve as a predictor of serum ALT and ALT levels at 24 hours after liver transplantation.

## Discussion

In the present study, we provide clinical evidence from two independent cohorts demonstrating a dramatic elevation in serum FGF21 levels as early as two hours after liver transplantation, well before the occurrence of massive hepatocellular damage. In addition, I/R injury-evoked elevation of serum FGF21 levels was coupled with the sharp increase in FGF21 protein levels in the liver. Furthermore, both the peak values and the amplitude of the increase in serum FGF21 levels in the first two hours significantly correlated with the magnitude of the elevation of serum levels of ALT and AST at day 1 post-reperfusion. These findings suggest that serum FGF21 can be potentially used as a sensitive and non-invasive biomarker for the early detection and prediction of hepatic I/R injury in patients with liver transplantation.

Our clinical data demonstrated a superior sensitivity of serum FGF21 compared to the currently used biomarkers (ALT and AST) for detection of hepatic I/R injury. The dramatic elevation of serum FGF21 levels occurs within the first 2 hours, whereas the peak of serum ALT and AST appears at 24 hours post-reperfusion. Furthermore, elevated serum level of ALT and AST is often the consequence of increased leakage of these enzymes from necrotic or apoptotic hepatocytes, occurring only after severe hepatocellular damage and cell death. By contrast, human FGF21 possesses a 29-amino-acid amino-terminal secretory signal sequence, which helps the protein to sort into the endoplasmic reticulum-golgi secretory pathways^17^. Therefore, FGF21 can be secreted into circulation to act as a metabolic hormone with pleiotropic effects on regulating glucose and lipid homeostasis and insulin sensitivity. In light of the dada demonstrating FGF21 as a secreted protein, I/R injury-induced elevation of serum level of FGF21 is mainly due to stress signal-induced significant augmentation of FGF21 expression in hepatocytes and the subsequent secretion into the circulation.

Another advantage of serum FGF21 over ALT and AST in the detection of liver injury is the tissue specificity. It is well-known that the non-hepatic source of serum ALT may inadvertently influence the decision on development of pharmaceutical compounds^7^,^8^,^9^. In the present study, our immunohistochemistry data showed that FGF21 protein levels in liver tissue were dramatically elevated upon hepatic I/R injury. Consistent with our clinical evidence, a recent study using FGF21 hepatocyte-specific knockout mice demonstrated that fasting-induced FGF21 mRNA expression in the liver and elevated FGF21 in circulation were completely abolished in hepatocyte-specific FGF21 knockout mice^33^, further supporting the notion that hepatocytes is the predominant contributor to circulating levels of FGF21. Although FGF21 expression has also been reported in other tissues, including heart^34^,^35^,^36^ and adipose tissue^37^, their contribution to circulating levels of FGF21 remains uncertain. Notably, our previous tissue profiling data in mice has shown that the enhancement of FGF21 expression resulted from drug-induced hepatotoxicity predominantly occurred in the liver, but not in pancreas, heart, kidney, epididymal white and brown adipose tissue, muscle and brain, supporting serum FGF21 as a specific biomarker for liver injury^31^. Taken in conjunction with a previous report indicating the liver as the predominant source for circulating FGF21^15^, FGF21 may exhibit better organ specificity for liver as compared with ALT; and may be potentially used for monitoring drug-induced acute liver injury in preclinical safety assessment.

We have observed high levels of serum ALT and AST in cohort-2 at 10 minutes after surgery. This elevation maybe due to cold storage ischemia occurring during donor organ preservation before transplantation^3^,^38^ Another peak of ALT and AST levels, which is observed at approximately 24 hours after transplantation, is likely to be caused by oxidative stress and immune cell activation during warm I/R injury^3^,^39^. Notably, there is a strong association between the magnitude of serum FGF21 elevation at 2 hours and increases of serum ALT level at 24 hours after transplantation, suggesting that serum FGF21 elevation may be attributed to warm ischemic injury after the return of oxygen delivery, but not cold storage ischemia. These findings further support the notion that distinct mechanisms are involved in ischemic injury at various stages of liver transplantation^39^; and also implicate that serum FGF21 may serve as a predictive indicator for the severity of liver I/R injury at the later phase of liver transplantation. Furthermore, the detection of blood FGF21 by immunoassay takes approximately 2 hours, suggesting that the assay for blood FGF21 is suitable and feasible in routine clinical use. According to our data, higher levels of blood FGF21 at early stage after liver transplantation indicate the increased risk for severe hepatic I/R injury. Therefore, patients with higher levels of FGF21 at early stage after liver transplantation should be under more close monitoring for primary graft non-function and primary graft dysfunction.

Although the pathophysiological role of FGF21 in hepatic I/R injury remains unclear at this stage, it is of interest to note that obvious liver damage (as determined by serum ALT and AST levels) occurs only after the dramatic elevation of serum FGF21 is markedly attenuated (Fig. 2A–D). These data raises the possibility that the marked induction of FGF21 expression may represent an adaptive and protective response, and the massive onset of cell death during liver I/R injury may be attributed in part to the decompensated elevation of serum FGF21. In support of this notion, FGF21 has been demonstrated to play a protective role in hepatotoxicity induced by acetaminophen, the leading cause of drug-induced liver injury^31^. In addition, the agonists of PPARα, a potent transcriptional activator of hepatic FGF21 expression, protect mice from acetaminophen-induced acute hepatotoxicity and I/R-induced liver injury through blocking oxidative stress and inflammation^40^,^41^. Collectively, these previous data in animal studies, together with our clinical findings, suggest that FGF21 not only acts as indicator for liver injury, but maybe also functionally involved in the pathogenesis of hepatic I/R injury.

Apart from hepatic I/R injury, the elevation in circulating levels of FGF21 has been demonstrated in patients with other diseases, including type 2 diabetes mellitus^42^,^43^,^44^, nonalcoholic fatty liver disease^45^, and atrial fibrillation^46^, although the magnitude of FGF21 elevation in these diseases are rather modest comparing to over 30-fold elevation in hepatic I/R injury. Thus, the elevation in blood FGF21 levels is not specific to liver disease, and other confounding factors affecting serum FGF21 levels should be considered as a limitation for the prediction of hepatic I/R injury. Another limitation of our study is the small sample size, and the relatively short follow-up period. The correlation between circulating FGF21 levels and clinical endpoint after liver transplantation cannot be determined at this stage. Therefore, this study is only of proof-of-concept in nature, and further investigations in large cohorts of different ethnic groups with long-term follow-up are needed to validate the diagnostic value of serum FGF21 for acute liver injury.

In summary, our present study has demonstrated that serum FGF21 is a sensitive biomarker for the detection of hepatic I/R injury in patients with liver transplantation. In light of the fact that the drastic elevation of serum FGF21 precedes the onset of hepatic damage, as suggested by elevated transaminase levels, measurement of this biomarker should be able to add both prognostic and mechanistic information to the current assessment of I/R injury during liver transplantation and other liver surgical procedures. Further investigations on the molecular pathways whereby liver transplantation causes the marked induction of FGF21 genes at the early stage, and the functional roles of FGF21 in the pathogenesis of hepatic I/R injury may help to design a more effective strategies for alleviation of the liver damage during transplantation.

## Methods

### Subjects and study design

Two independent cohorts of liver transplantation patients were recruited for this study. The design for the first cohort 1 is a retrospective cohort study. The clinical characteristics of the patients in these two study cohorts are described in Table 1. The study design for the first cohort is a retrospective cohort study. A total of 38 patients who received liver transplantation at the Department of Surgery, Queen Mary Hospital, The University of Hong Kong, were recruited for the determination of FGF21 levels in blood samples collected at 2 hours after hepatic artery declamping. Liver biopsies were collected before and 2 hours post implantation of donor liver. Age- and sex-matched healthy subjects were retrospectively chosen from Shanghai Community study as we reported previously^45^. For the second study cohort, a total of 13 liver transplantation patients were prospectively enrolled from the Division of Liver Transplantation, Shanghai Zhongshan Hospital, Fudan University, China. Blood samples were drawn from arteries through an indwelling radial artery catheter at various time intervals. Written consents were obtained from all subjects. Both studies were carried out in accordance with the guidelines approved by human ethics committees in The University of Hong Kong and Fudan University, respectively.

### Immunoassays and Biochemical Analysis for Serum Samples

Circulating concentrations of FGF21 were determined with commercially available human FGF-21 immunoassay kits, which are specific for the detection of human FGF21 (Antibody and Immunoassay Services, Hong Kong, China), as we described previously^31^,^47^,^48^. In brief, human FGF21 immunoassay is a quantitative enzyme-linked immunosorbent assay (ELISA). The 96-well plate was pre-coated with a rabbit polyclonal antibody specific for human FGF-21. Standards prepared by recombinant human FGF21 protein which was produced from by *E. Coli* (host strain BC21 (DE3)) and samples were pipetted into the wells for incubation. Afterwards, a biotin-labelled polyclonal antibody specific for human FGF-21 was added to the wells. After incubation with streptavidin-HRP conjugate, an HRP substrate solution was added for colour development. Since the increases in absorbance are directly proportional to the amount of captured human FGF-21, the unknown sample concentration can be interpolated from a standard curve included in each assay. The assay range of human FGF21 kits is 30–1920 pg/ml. Before the chemical assay, blood FGF21 levels were tested to be stable after two to three freeze-thaw cycles with the coefficients of variation (CV) being 8.1%. Two in-house controls (high- and low-level controls) were run in each assay. The intraassay CV was 1.3% and 1.7% for low-level controls (90 pg/ml) and high-level controls (450 pg/ml), respectively. The interassay CV was 5.5% and 2.6% for low-level controls and high-level controls, respectively. The reproducibility of the human FGF21 immunoassay was consistent with the previous report using the same kits^49^. FGF21 immunoassay in blood samples were conducted by experienced technician, who is blinded to the grouping information for all the blood samples. Serum activities of ALT and AST were measured with a commercial kit (Sigma-Aldrich, USA). All the biochemical assays were done in duplicate. Biochemical parameters in human blood samples were analyzed on the automated Roche Modular DDP Analyzer (Roche Diagnostics, USA).

### Histological Analysis and Immunohistochemistry

Liver specimens collected before or 2 hours post-implantation were fixed in 10% formalin solution (Sigma-Aldrich, USA); and paraffin-embedded liver sections were stained with hematoxylin-eosin (Sigma-Aldrich, USA). For FGF21 immunohistochemical staining, deparaffinized and dehydrated liver sections were incubated with an affinity-purified rabbit antibody against FGF21 (Antibody and Immunoassay Services, Hong Kong, China) in a solution containing 3% bovine serum albumin overnight at 4 °C, followed by the reaction with a horseradish peroxidase-conjugated secondary antibody against rabbit IgG (Cell Signaling Technology, USA) and 3,3′-Diaminobenzidine tetrahydrochloride (Sigma-Aldrich, USA). Immuno-stained slides were visualized with an Olympus biological microscope BX41, and images were captured with an Olympus DP72 color digital camera.

### Statistical Analysis

All analyses were performed with Statistical Package for Social Sciences version 14.0 (SPSS, Chicago. IL). Data were expressed as mean ± S.D. Statistical significance was determined by one-way ANOVA. Correlations between serum FGF21 levels and biochemical variables were analyzed with Pearson correlation. Receiver operating characteristics (ROCs) were calculated to explore the diagnostic performance of circulating FGF21 to predict severe hepatic I/R injury post liver transplantation (defined by serum levels of AST at day 1 post liver transplantation >240 U/L (6 times upper limit of normal)). The closer the ROC curve is located in the left upper quadrant of the graph, the more accurate is the test variable because the true-positive rate (sensitivity) approximates 1.0 and the false positive (1-specificity) rate approximates 0. The optimal cut-off value of serum FGF21 for predicting severe hepatic I/R injury was established by Youden index (*J*), which was calculated as *J* = maximum {sensitivity + specificity −1}^50^. The *P* value (*P* < 0.05) reflects a significantly statistical difference of the results obtained toward the null hypothesis (area under the ROC curve is 0.5). In all statistical comparisons, a *P* value < 0.05 was used to indicate a statistically significant difference.

## Additional Information

**How to cite this article**: Ye, D. *et al*. Circulating Fibroblast Growth Factor 21 Is A Sensitive Biomarker for Severe Ischemia/reperfusion Injury in Patients with Liver Transplantation. *Sci. Rep.* **6**, 19776; doi: 10.1038/srep19776 (2016).

## Figures and Tables

**Table 1:** Demographic and clinical data in clinical study cohort-1 and cohort-2.

	Healthy controls (n = 38)	Study cohort-1 (n = 38)	Study cohort-2 (n = 13)
Gender (male : female)	30: 8	30: 8	9: 4
Age (yr)†	52.6 (40–69)	53.5 (40–64)	53.0 (43–63)
HBV positive	—	31 (81.6%)	10 (76.9%)
Primary disease (HCC: others)	—	26: 12	12: 1
Median ALT before LTx (U/L)*	22.5 ± 1.2	44.5 ± 380.9	66.9 ± 50.1
Median AST before LTx (U/L)*	19.2 ± 1.7	66.5 ± 257.9	50.2 ± 43.9
Median AFP before LTx (ng/mL)†	—	10.0 (1–6040)	116.2 (2.8–8232)

Abbreviations: HBV, hepatitis B virus; HCC, hepatocellular carcinoma; AFP, alpha-fetoprotein; LTx, liver transplantation.

^†^Data are expressed as medians (range).

^*^Data are expressed as mean ± S.D. −, not available.
